# sEMG-Based Motion Recognition of Upper Limb Rehabilitation Using the Improved Yolo-v4 Algorithm

**DOI:** 10.3390/life12010064

**Published:** 2022-01-03

**Authors:** Dongdong Bu, Shuxiang Guo, He Li

**Affiliations:** 1 Key Laboratory of Convergence Biomedical Engineering System and Healthcare Technology, The Ministry of Industry and Information Technology, School of Life Science, Beijing Institute of Technology, No. 5, Zhongguancun South Street, Haidian District, Beijing 100081, China; budongdong@bit.edu.cn (D.B.); lihe@bit.edu.cn (H.L.); 2 Faculty of Engineering, Kagawa University, 2217-20 Hayashi-cho, Takamatsu 760-8521, Kagawa, Japan

**Keywords:** surface electromyography, upper limb motion recognition, feature extract network, target detection network, improved Yolo-v4 algorithm

## Abstract

The surface electromyography (sEMG) signal is widely used as a control source of the upper limb exoskeleton rehabilitation robot. However, the traditional way of controlling the exoskeleton robot by the sEMG signal requires one to specially extract and calculate for complex sEMG features. Moreover, due to the huge amount of calculation and individualized difference, the real-time control of the exoskeleton robot cannot be realized. Therefore, this paper proposes a novel method using an improved detection algorithm to recognize limb joint motion and detect joint angle based on sEMG images, aiming to obtain a high-security and fast-processing action recognition strategy. In this paper, MobileNetV2 combined the Ghost module as the feature extraction network to obtain the pretraining model. Then, the target detection network Yolo-V4 was used to estimate the six movement categories of the upper limb joints and to predict the joint movement angles. The experimental results showed that the proposed motion recognition methods were available. Every 100 pictures can be accurately identified in approximately 78 pictures, and the processing speed of every single picture on the PC side was 17.97 ms. For the train data, the mAP@0.5 could reach 82.3%, and mAP@0.5–0.95 could reach 0.42; for the verification data, the average recognition accuracy could reach 80.7%.

## 1. Introduction

Upper limb movement is very central for daily activities; however, there are about 15 million people a year who suffer from stroke worldwide, with 5 million stroke survivors who experience permanent motor disability and require therapeutic services [1]. Early intervention in rehabilitation therapies has a desirable effect for the recovery of patients [2]. The robot exoskeleton can assist hemiplegia patients with pretraining their own rehabilitation exercise, guide the correct track to perform the movement and give the support of the auxiliary force directly. To improve the efficiency of the whole rehabilitation process, there are many technical difficulties in the field of exoskeleton robotics. Therefore, how to make the exoskeletons work as flexibly as a real limb is now a key point [3,4,5]. The sEMG was a reliable way that has been widely applied to rehabilitation assessment [6,7], human intention prediction [8,9,10], human motion classification [11,12,13], prosthesis control [14], and rehabilitation robot control [15,16,17,18,19]. Miao et al. [20] presented a platform robotics system with a subject-specific workspace for bilateral rehabilitation training. Y. Liu et al. [21] presented a novel bilateral rehabilitation system that implements a surface electromyography (sEMG)-based stiffness control to achieve real-time stiffness adjustment based on the user’s dynamic motion. Z. Yang et al. [22] proposed a mirror bilateral neuro-rehabilitation training system with sEMG-based patient active participation assessment that was proposed for the bilateral isometric force output coordination of the upper limb elbow joint. With the mirror visual feedback of the human–robot interface, the hemiplegia patients could perform bilateral isometric lifting tasks with modulated robotic assistance intuitive cognition of motor control of bilateral limbs.

sEMG signals have verified that there is good performance for the human intention prediction because sEMG signals can immediately reflect the muscle activity and movement intention [23]. In general, the basic process of sEMG-based human intention prediction includes data preprocessing, data segmentation, feature extraction and classifier design [24]. The action-recognition accuracy, as well as the real-time and model robustness, are very important factors during the process of rehabilitation training. For the action recognition accuracy, multi-channels sEMG acquisition technology and feature extraction methods are available to improve the action recognition accuracy. Multi-channels sEMG can capture high spatial and temporal resolution signals, which is the most commonly used data acquisition technology in muscle force estimation and limb motion analysis [25]. Although multi-channel sEMG can collect abundant information to improve recognition accuracy, there are also the problems of information redundancy and inter-channel crosstalk. Meanwhile, the good feature extraction methods can improve the performance for the muscle activation analysis, muscle strength estimation, motion pattern analysis and so on [26] in practical applications. However, it needs to be processed deeply to extract the most appropriate sEMG features and the huge amount of calculation. F.Y. Xiao et al. [27] proposed the sEMG encoding and feature extraction methods, GADF-HOG method and GASF-HOG method, for human gesture recognition. They were inspired by image neurons and combines with computer graphics processing technology to extract sEMG image features. The results verified the feasibility of finding possible solutions from brain science and computer science to extract sEMG features and apply them to gesture recognition. For the model robustness, non-stationary characteristics of sEMG is the most critical impact factor in the accuracy and reliability of the recognition algorithm, which increases the uncertainty of the motion recognition algorithm [28]. Therefore, a trained model for a specific user cannot be applied to another user because of individual differences among the subjects, muscle parameters, including muscle thickness, and innervation zone location. In order to reduce the impact of non-stationary characteristics of sEMG, feature engineering was applied to improve the robustness of the algorithm [29]. Spanias et al. [30] applied a log-likelihood metric to detect the sEMG disturbances and then to decide whether to use it or not. When sEMG contained disturbances, the classifier would detect the disturbances and disregard sEMG data. S.H. Qi et al. [31] proposed a new initiative via deep learning to recognize general composite motions, which processes sEMG signals as images. A well-designed convolutional neural network (CNN) was used to obtain effective filters automatically to extract features. The reliability of the recognition algorithm could be ensured by giving up those unqualified sEMG signals. However, it was not available to discard any existing signal for the accuracy of the algorithm. Although there were differences in sEMG distribution for different subjects, it still contained motion information, especially for conventional low-density electrodes.

Considering the action-recognition accuracy and model robustness, the purpose of this paper is to achieve a real-time target detection model for the upper limb motion classification and motion angle prediction with high accuracy and reliability, especially for conventional low-density electrodes. Firstly, the multi-channel sEMG signal data was reduced through the classification network model, and the signal channels with high classification accuracy were retained. Next, the reduced signal channels were weighted and summed according to the importance of effective information to realize channel fusion, and then the fused sEMG features were extracted; a lightweight MobileNet-V2 combined with the Ghost module was used as a backbone network to extract features based on sEMG images; finally, the improved Yolo target detection network was used to realize the motion pattern recognition and motion amplitude detection of wrist and elbow based on sEMG signal to effectively reduce the impact of the non-stationary sEMG signal and improve the reliability and accuracy of the prediction.

## 2. Methods

### 2.1. System Overview

**Figure 1:**
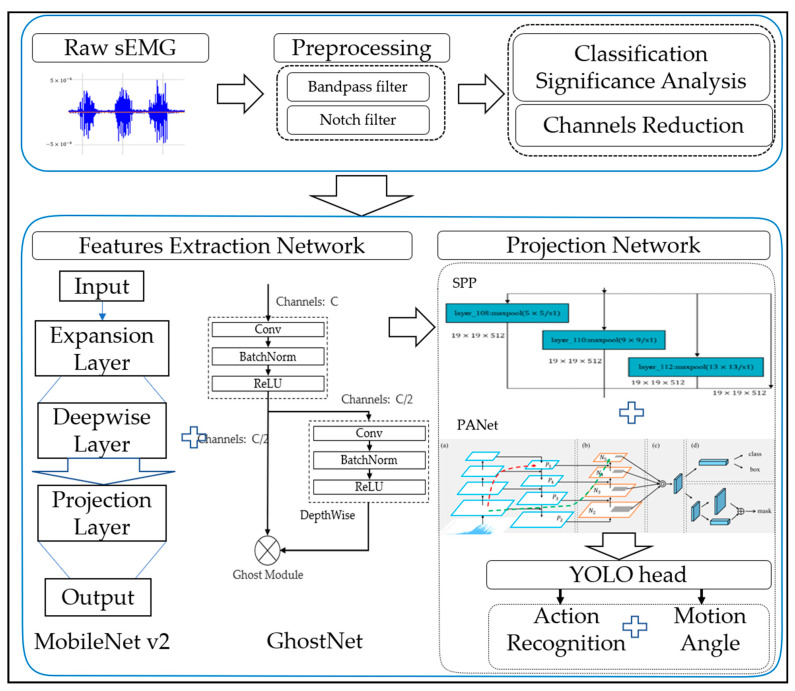
An overall diagram of the subject-independent gesture recognition method based on a transferred learning model.

Figure 1 presents the overall diagram of the proposed method, which is mainly divided into five parts: sEMG acquisition, preprocessing of sEMG signal, classification significance analysis and channels reduction, feature extraction network of sEMG signal, and gesture classification and joint motion maximum angle prediction. Specific details of corresponding parts are introduced below.

### 2.2. sEMG Acquisition

**Figure 2:**
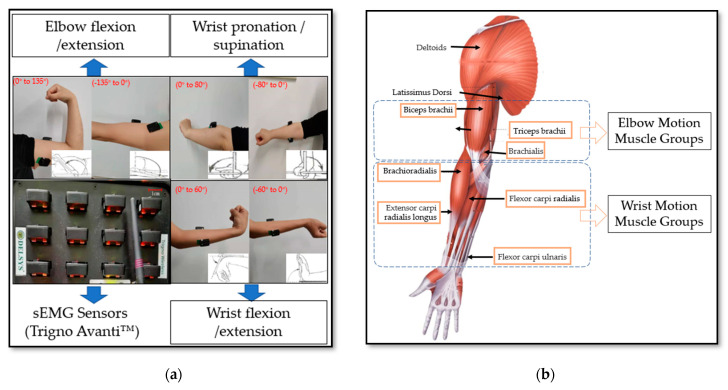
sEMG acquisition sensors and muscle groups related to joint movement. (**a**) Electrode placements for sEMG data acquisition and the acquisition range of joint motion; (**b**) Human upper limb muscle diagram and the upper limb motion muscle groups.

In this paper, three healthy subjects were recruited. Some instructions on the data acquisition were known to subjects in advance, thus ensuring the consistency of the performing actions and the rationality of the data analysis. Before the experiment, the skin of the subjects’ corresponding muscles was wiped with alcohol. As shown in Figure 2a, the sensors for signal acquisition were attached to the subject’s left upper limb to record different classification sEMG signals and joint angles of the upper limb. The signal acquisition equipment, Trigno Avanti™, was exploited to acquire the sEMG signals, as presented in Figure 2a. The device is a wireless physiological data acquisition and analysis system, which can record 16 sEMG data channels at the same time, with a sampling rate of 2000 Hz for each channel. The sEMG signals were recorded for six selected types of human upper limb action via Trigno Avanti™, which include elbow flexion/extension, wrist flexion/extension, wrist pronation/external rotation. The sEMG sensors were attached to the corresponding muscle group of joint movement, including biceps brachii, triceps brachii, extensor carpi radialis, and flexor carpi radialis and so on. Each motion data was recorded for 5 s, 10 times in total, which sets a group; there were 8 groups for each person. There was a 5-min interval between each movement collection to prevent muscle fatigue. Due to the non-steady characteristics of the sEMG signal, the signal acquisition process used the controlled variable method, strictly stipulating the acquisition time, the acquisition time of each type of action, and the interval time of each group of actions. In order to ensure the consistency of the collected signals, the collection time of each subject was set from 9 a.m. to 11 a.m. The purpose of this paper was to train a real-time target detection model to predict the movement category and range of upper limbs at the same time, so the predicted movement category and joint movement range are shown in Figure 2a. The human upper limb muscle diagram is shown in Figure 2b, and the muscle groups involved in the movement of the elbow and wrist are shown in Figure 2b.

### 2.3. sEMG Signal Preprocessing

**Figure 3:**
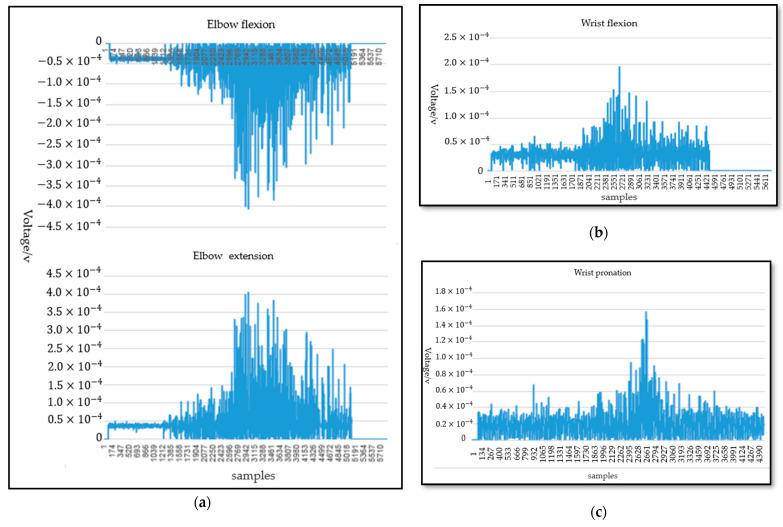
The sEMG signal distribution. (**a**) The sEMG signal distribution of elbow joint flexion/extension; (**b**) The sEMG signal distribution of wrist joint flexion; (**c**) The sEMG signal distribution of wrist joint pronation.

The influence of noise was very significant in the process of sEMG signal acquisition. The energy of the EMG signal was mainly concentrated at 20–450 Hz according to the energy density spectrum of the EMG signal. sEMG signals with energy less than the electrical noise level are ignored for signals outside the frequency range of 20–450 Hz. Therefore, a band-pass filter of 20–450 Hz and 50 Hz notch filter are applied to eliminate the noise and the artifacts. The sEMG signals were further digitized after eliminating the noise in the raw sEMG signals. The waveform of the sEMG signals oscillates randomly and frequently around zero amplitude. When the subject performed an action, the change of amplitude of the sEMG was more obvious and easier capture intuitively. Therefore, the analysis of movement separability based on sEMG was necessary. The data for each subject from 2 s to 4 s were intercepted because of the instability of movement during acceleration and deceleration. This paper studies the sEMG signals of six types of motions with three degrees of freedom at the elbow and wrist joints. As shown in Figure 3a, under the same acquisition scheme, the data distribution of sEMG for elbow joint flexion/extension was similar; however, the amplitudes were opposite to each other. Then, the data distribution of sEMG for wrist pronation/external rotation and wrist joint flexion/extension was similar to the elbow joint flexion/extension. The data distribution of sEMG for wrist joint pronation and elbow joint flexion is shown in Figure 3b,c. Enough data was needed for a deep neural-network training model. Therefore, data augmentation was necessary to prevent the overfitting of the model. Data augmentation enhances the size and quality of training datasets, intending to achieve better generalization. In this paper, horizontal flipping, image displacement or clipping, color dithering, and simultaneous rotation, and random scale transformation are applied.

**Figure 4:**
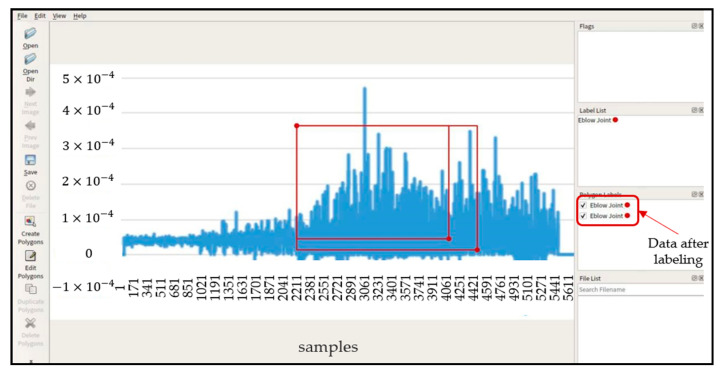
Data labeling method.

SEMG images and motion sequences of different types of tasks need different data for labeling. Human upper limb motion intentions generally included joint angle, movement patterns, and functional movement. Data labeling is very important in the upper limb motion intentions. In this paper, the rectangle labeling method was applied to label the input data, as shown in Figure 4. During the labeling process, the data labeling tool will generate corresponding “. JSON” files to store the coordinates of the midpoint of the target frame of the image, the storage address of the picture data, the distance from the upper boundary of the target frame to the center point and the distance from the left boundary to the center point in the picture data.

### 2.4. sEMG Channels Reduction

The signal channel screening of the sEMG signal is a very important task. Too many channels can retain more information of action parts; however, the corresponding disadvantage is that too much information will lead to redundancy, affect the computing power of the recognition network, increase the reasoning burden, and affect the recognition efficiency and time of the network model, Therefore, it was not desirable to reserve too many signal channels. To sum up, the screening of the sEMG signal acquisition channel is an indispensable step. Although the signal channel loses some effective information and physiological characteristics and reduces the accuracy of action recognition, it will improve the training and reasoning speed of the network and reduce the memory occupation of the action-recognition network. It can not only realize the online real-time recognition function, but also facilitate the deployment of the model because of the reduction of the pretraining model. The lightweight network MobileNetV2 was applied to determine whether the information provided by the channel was useful. The comparative experiment was performed and the channel reduction of the sEMG signal was realized according to the analysis of the accuracy and processing speed of the experimental results.

For the reduction of elbow joint sEMG signal channels, the muscle groups involved in the movement of elbow joint mainly included biceps brachii (main channel), triceps brachii (secondary main channel) and brachialis. As shown in Table 1, three groups of comparative experiments were performed with biceps brachii, biceps brachii and triceps brachii, biceps brachii, triceps brachii and brachialis, respectively. Each scheme consisted of 1600 train data and 200 verification data. According to the comparative experimental results, in the process of elbow movement, the number of channels is positively correlated with the accuracy of model recognition and negatively correlated with the time-consuming of model processing data on the test set. Table 1 shows the results of the three experimental schemes. The biceps brachii carries relatively rich EMG signals; the triceps brachii also carries some effective information. Therefore, the biceps brachii and triceps brachii channel signals were retained in this paper.

For the reduction of wrist joint sEMG signal channels, the muscle groups involved in the movement of the wrist joint mainly included Extensor carpi ulnaris, Flexor carpi ulnaris, Extensor carpi radialis and Flexor carpi ulnaris. Three groups of comparative experiments were performed. Each scheme consisted of 1600 train data and 200 verification data. As shown in Table 2 and Table 3, according to the comparative experimental results, extensor carpi ulnaris was the main muscle active area for the wrist flexion/extension and the extensor carpi radialis is the main muscle active area for wrist pronation/external rotation. The number of channels was positively correlated with the accuracy of model recognition but negatively correlated with the time-consuming model processing data on the test set. Therefore, considering the accuracy and time-consuming of the model, extensor carpi ulnaris and extensor carpi radialis were retained in this paper. Finally, sEMG signals were collected from four muscle groups synchronously.

### 2.5. Feature Extraction Network Model

The effectiveness and good performance have been verified for the convolution neural network (CNN) in feature extraction of sEMG signals. Considering the size of the network model and the speed of model inference when the network model was deployed, the MobileNetV2 combined with the Ghost module [32,33] (MGNet) was applied to extract the feature map of sEMG signal. The feature extraction network includes two outputs: the compressive feature and the reconstructive sEMG signal.

**Figure 5:**
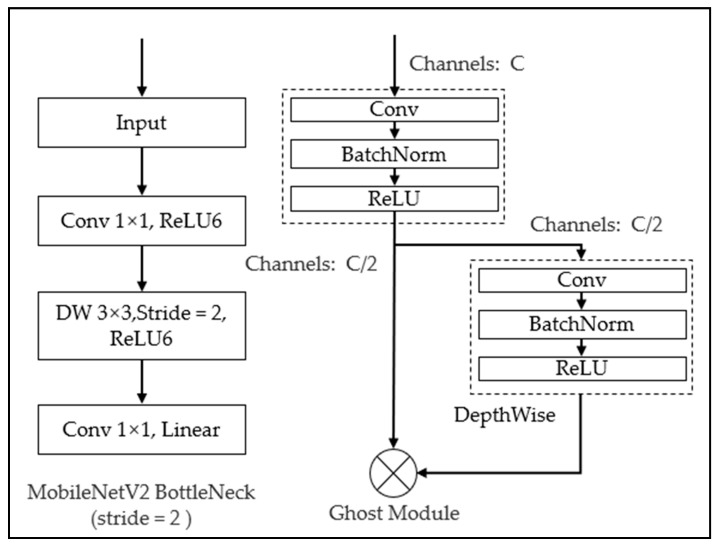
The bottleneck of MobileNetV2 architecture with stride = 2 and the Ghost Module.

The inverse residual structure was used in MobileNetV2. One bottleneck of MobileNetV2 architecture and the Ghost module is shown in Figure 5. Firstly, the 1 × 1 convolution kernel was applied to dimension upgrading of sEMG data based on the previous bottleneck. Then, the conventional convolution was used to extract features. Finally, the 1 × 1 convolution kernel was reused for data compression. Based on the conventional MobileNetV2 architecture and the Ghost module, an improved feature extraction method of sEMG signals was proposed in this paper. Therefore, there are two improvements to the MobileNetV2 structure. Firstly, for the block loop module, when the number of bottleneck modules in the MobileNetV2 structure exceeded three times, the Ghost module was introduced for identity mapping and linear connection to replace the block cycle structure. Secondly, for the loss function, during the upper limb movement, the horizontal width loss and vertical height loss of the sEMG feature image were added to the original loss function of MobileNetV2. The input of MGNet was labeled sEMG images with the size of 448 × 448. In this paper, six classes of sEMG signal images were divided into 90,000 training data with 15,000 for each type of action, 15,000 test data with 2500 for each type of action, 900 verification data with 150 for each type of action. The category labels use Arabic numerals from 1 to 6 to mark joint movements; wrist flexion/extension uses 1 and 2, wrist pronation/supination uses 3 and 4, and elbow flexion/extension uses 5 and 6.

To obtain a feature extraction network model with good performance, the data label array (label, sEMG action width, action peak) included action category, joint action label-rectangle width and surface EMG signal peak. In this paper, the feature extraction network structure took the bottleneck module of mobilenetv2 as the basic module as the framework and replaces the pooling layer with the convolution kernel with step size string = 2. When the bottleneck module was repeated three times, the Ghost module was introduced. The Ghost module divided the input EMG into parts. The first part used the conventional DeepthWise (DW) convolution module and the second part used the cheap-operation module. The two modules were convolution layers, BatchNorm normalization and nonlinear activation layer, respectively. The difference is that the convolution kernel size in the convolution layer was different and the kernel size in the first stage was 1 × 1. The kernel size in the second stage was 3 × 3 and the step size at the end of the Ghost module repetition times would generate 64 channels through the expansion factor, which was composed of DW convolution module and cheap operation module, which provides a characteristic diagram of half the number of channels. The remaining layers are composed of bottleneck modules, and the size of the feature map was also controlled by the step size. As shown in Table 4 and Table 5, the network structure and parameters quantity of MGNet feature extraction network were listed. The super parameter settings of MGNet included learning rate = 0.0003 and training batch_size = 16, training times Max_ Epochs = 3000; the parameter attenuation rate was 0.5 times without change for 3 consecutive epochs and they are saved every 200 training times. For the loss function of MGNet, the overall loss of MGNet was divided into two parts, which are CrossEntropyLoss and Manhattan distance from the start position of action amplitude of the sEMG signal to the end position of action (the horizontal width loss of sEMG signal $l_{w}$) and from the peak value of sEMG signal amplitude to the starting axis when the action occured (vertical loss of action amplitude $l_{h}$). The MGNet loss function was calculated as represented in (1).

(1) $$Loss_{MGNet}=\mathrm{CrossEntropyLoss}+\alpha l_{w}+\beta l_{h}$$

where α and β are the horizontal width loss coefficient and vertical amplitude loss coefficient of ground EMG signal, respectively. In this paper, α=0.13 and β=0.26. After the feature extraction and feature mapping layer of the raw sEMG signal image, the output image size of MGNet was 7 × 7. The full connection layer was replaced by the average-pooling layer. Finally, 1 × 1 convolution was applied to motion classification.

### 2.6. The Prediction Network

The primary function of the prediction network was to predict the future motion of humans. An improved target detection algorithm Yolo-v4 [34] was applied to achieve the goal. The output of prediction network consisted of two independent parts, corresponding to the movement category of human limb and maximum joint motion angle prediction.

**Figure 6:**
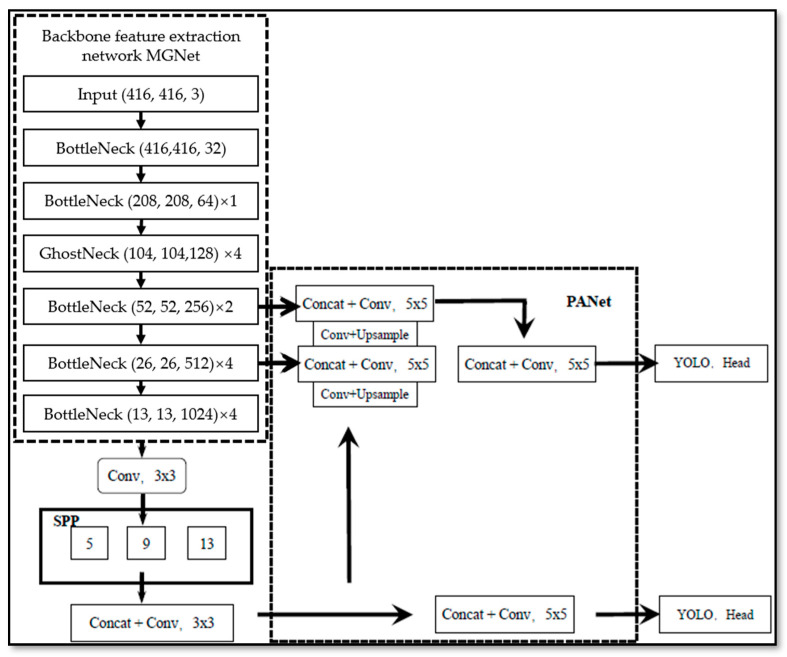
Proposed Yolo-v4 network model structure.

Figure 6 shows the architecture of the prediction network. Based on the traditional Yolo-v4 algorithm, there were three improved parts for the prediction network: for the loss function, the aspect ratio of sEMG feature images was added; The number of path aggregation network (PANet) layers was adjusted; mosaic data enhancement was not used.

**Figure 7:**
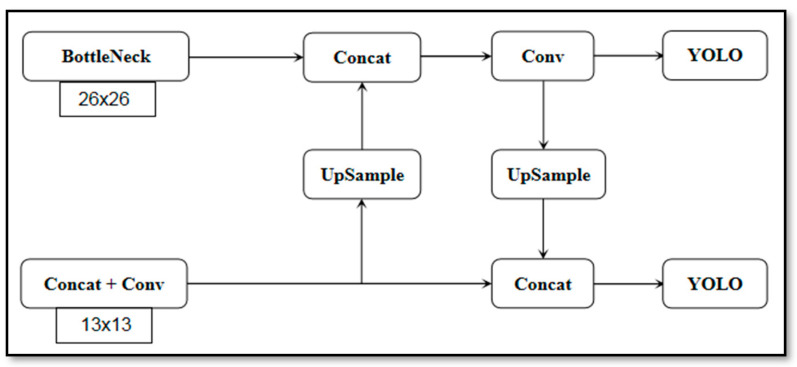
Improved PANet microstructure diagram.

For the PANet in the process of data processing [33], small grid detection was used for large targets; large grid detection was used for small targets. The input of PANet was directly obtained from the convolution layer of the backbone feature extraction network, spliced with the feature layer through the down-sampling operation, as shown in Figure 7; the convolution operation was then used to complete the feature map fusion between different layers. In this paper, PANet was used to 26 × 26 and 13 × 13 feature maps for feature fusion.

**Figure 8:**
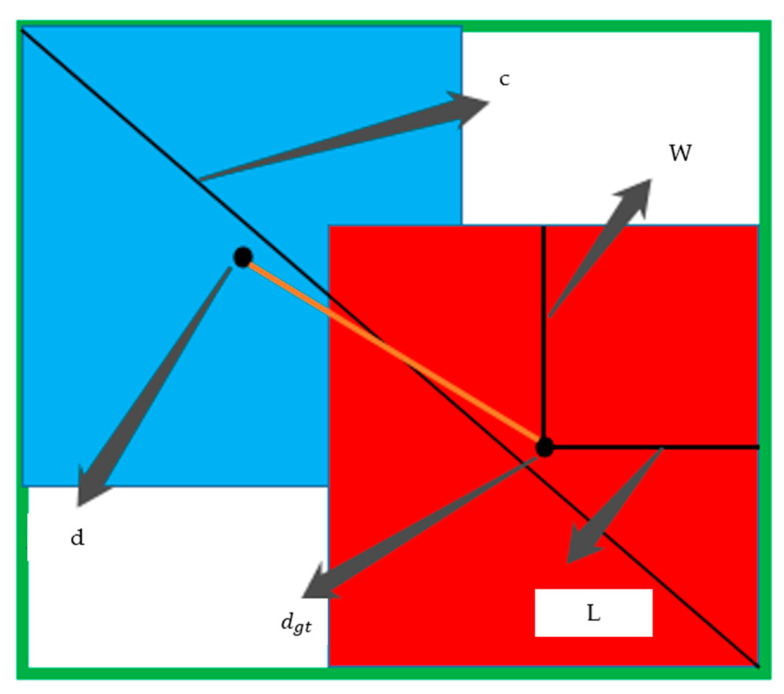
The loss function of the target detection prediction network.

For the target detection, prediction network, the loss function must include three kinds of information: overlapping area, center point distance and aspect ratio to avoid the gradient disappearance in the process of backpropagation. Based on the loss function of Yolo-V4, the aspect ratio of sEMG feature images was introduced, as shown in Formula (2).

(2) $$Loss_{dec}=1-IoU+\frac{\rho^{2}\left(d,d_{gt}\right)}{c^{2}}+\left(\frac{L_{gt}}{W_{gt}}-\frac{L}{W}\right)$$

where 1−IoU represents the size of the non-overlapping area between the real target frame and network prediction frame, as shown in Figure 8. When the intersection of two boxes became larger and larger, the overlapping area would increase and the value of  1−IoU would become smaller and smaller. $\rho^{2}\left(d,d_{gt}\right)$ represents the Euclidean distance between the center point of the real target frame and the center point of the network prediction frame. d and $d_{gt}$ represent the center points of the network prediction frame and the real target frame location, respectively. c represents the distance from the upper left corner to the lower right corner after the real target frame coincided with the network prediction frame. L and W represents the distance from the center point of the prediction box to the left boundary and the distance from the center point of the prediction box to the upper boundary. Similarly, $L_{gt}$ and $W_{gt}$ are the real distance. To prevent missed detection due to the strong filtering candidate box and the ability of non-maximum-suppression (NMS) in the prediction stage, soft NMS is applied to the target detection prediction network. The cross-stage local network can improve the learning ability of the feature extraction network, reduce the calculation and reasoning time and reduce the occupied memory. Mosaic data enhancement was not be used in this paper, because the effect of using mosaic data enhancement in the process of network training was not effective, and even leads to the decline of accuracy.

### 2.7. The Evaluation Criteria

**Figure 9:**
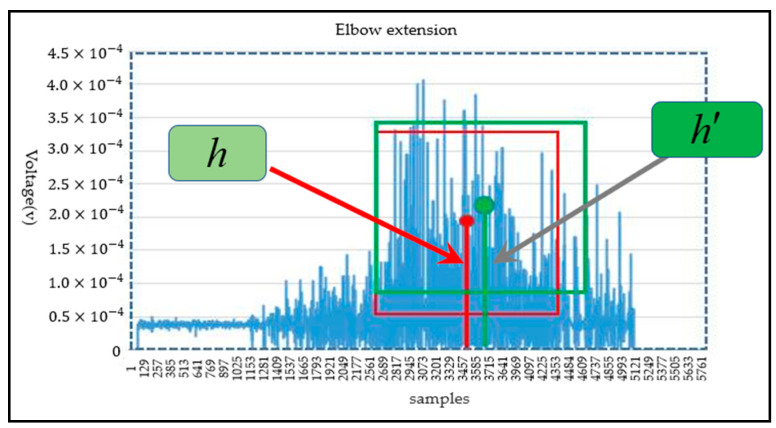
The evaluation rules of joint motion angle for target detection.

In this paper, for the evaluate of motion classifications, mean average precision (mAP) is used to evaluate target detection network performance as shown in Formula (3). mAP can be used to evaluate quality of the learned model in all motion classifications.

(3) $$mAP=\frac{1}{m}\sum_{i=1}^{m}AP_{i}$$

where m represents the number of recognition categories. $AP_{i}$ represents the average precision (area under precision and recall curves) value of the ith action. mAP@0.5 represents the mean average precision when the intersection union ratio (IoU) is 0.5. mAP@0.5–0.95 corresponds to the average AP for IoU from 0.5 to 0.95, with a step size of 0.05. The loss curve for each data set of the Yolo-V4 model was applied to reflect the accuracy of classification. For the evaluation of joint motion angle in the target detection network; the network model output of the Yolo-v4 algorithm includes the coordinates of the center point of the prediction frame, the length of the distance from the upper boundary of the prediction frame to the midpoint, and the width from the left boundary of the prediction frame to the center point. The ordinate value of the center point coordinate of the prediction frame represents the voltage of the corresponding motion of the joint; however, the center point coordinate, the length of the distance from the upper boundary of the prediction frame to the midpoint and the width from the left boundary of the prediction frame to the center are relative positions. In this paper, the center point position of the target frame was the maximum angle range corresponding to the movement of the upper limb joint. As shown in Figure 9, for the joint angle prediction of the elbow joint extension motion, the blue dashed box was the input image size; the red box was the target real box; the ordinate value of center point of real frame was h, representing the maximum motion angle of the joint. The green frame was the prediction frame; the ordinate value of the prediction frame center point was $h^{\prime }$. If $\left|h^{\prime }\right|>\left|\;h\;\right|$, the predicted motion joint angle of elbow extension movement is the maximum angle of the real frame. Thus, the prediction angle was predicted as the maximum motion angle of the real joint by the prediction model; if $\left|h^{\prime }\right|<\left|\;h\;\right|$, the prediction angle was calculated according to Formula (4), where variables $\;C_{pre\_angle}$ and $C_{\max\_angle}$ represent the prediction motion joint angle and the maximum motion angle of the real joint respectively.

(4) $$C_{pre\_angle}=\{\begin{array}{c} \frac{h^{\prime }}{h}\times C_{\max\_angle}\;\;\;\;\;\;\;\;\;\;\;\;\;\;\;\;if\;\left|h^{\prime }\right|<\left|h\right|\\ C_{\max\_angle}\;\;\;\;\;\;\;\;\;\;\;\;\;\;\;\;\;\;\;\;\;if\;\left|h^{\prime }\right|>\left|h\right| \end{array}\;\;\;\;\;$$

## 3. Results

The motion prediction system consisted of feature extraction and motion prediction. The goal of the motion prediction system was to obtain reliable and precise human motion intention. The performance of each part was verified respectively.

### 3.1. The Performance of Feature Extraction Network

**Figure 10:**
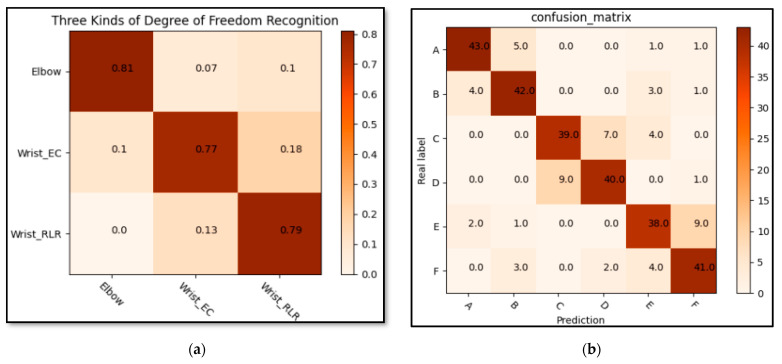
Confusion matrix of feature network recognition: (**a**) Confusion matrix of three degrees of freedom recognition of upper limb; (**b**) Confusion matrix of six kinds of upper limb movements.

The goal of feature extraction was to obtain the compressed sEMG feature. Firstly, the correlation of sEMG signals among the three degrees of freedom was analyzed; each degree of freedom of the marked data was set into a class to identify the correlation of degrees of freedom. The purpose was to judge whether the experimental scheme was reasonable according to the correlation between degrees of freedom. Figure 10a gives a confusion matrix for the identification of three degrees of freedom. The confusion matrix was verified and drawn through the feature extraction network model. As shown in Figure 10a, the label of elbow motion was elbow and the label of wrist extension/contraction freedom was wrist_ EC; the label of wrist left/right rotation was wrist_ RLR; the error rate between the two degrees of freedom of the wrist was significantly higher than that of the elbow, but the error rate was still within the acceptable range (less than 0.2). However, due to the different proportion of different signal channels and the corresponding signal amplitude of action amplitude, the signal characteristics were also different; thus, the separability of three degrees of freedom action was analyzed by comparative experiment. Through the prediction results of the trained MGNet on the verification set, the inter-class average recognition accuracy was 0.7. According to the above experimental results, it can be found that different degrees of freedom were obviously separable. Then the sEMG signal diagrams of six types of actions were verified by MGNet. In this paper, the classification performance was compared among MGNet, MobileNet V2 and GhostNet, as shown in Table 6. For the test data, the prediction performance of the classification model and the time spent processing the same number of pictures were compared. The experiment also drew the confusion matrix according to the classification results of the verification set, as shown in Figure 10b. Fifty randomly selected pictures of each action on the verification data and the confusion matrix were drawn according to the MGNet prediction label and real label. According to the experimental results, the memory occupied by the training model of the feature extraction network will be less than 1.9 M, the reasoning and prediction speed will be 107.3927 s, and 15,000 pictures can be processed. According to the above experimental results, it can be judged that the sEMG signal diagrams of three degrees of freedom and six actions were significantly separable.

### 3.2. The Performance of Prediction Network

**Figure 11:**
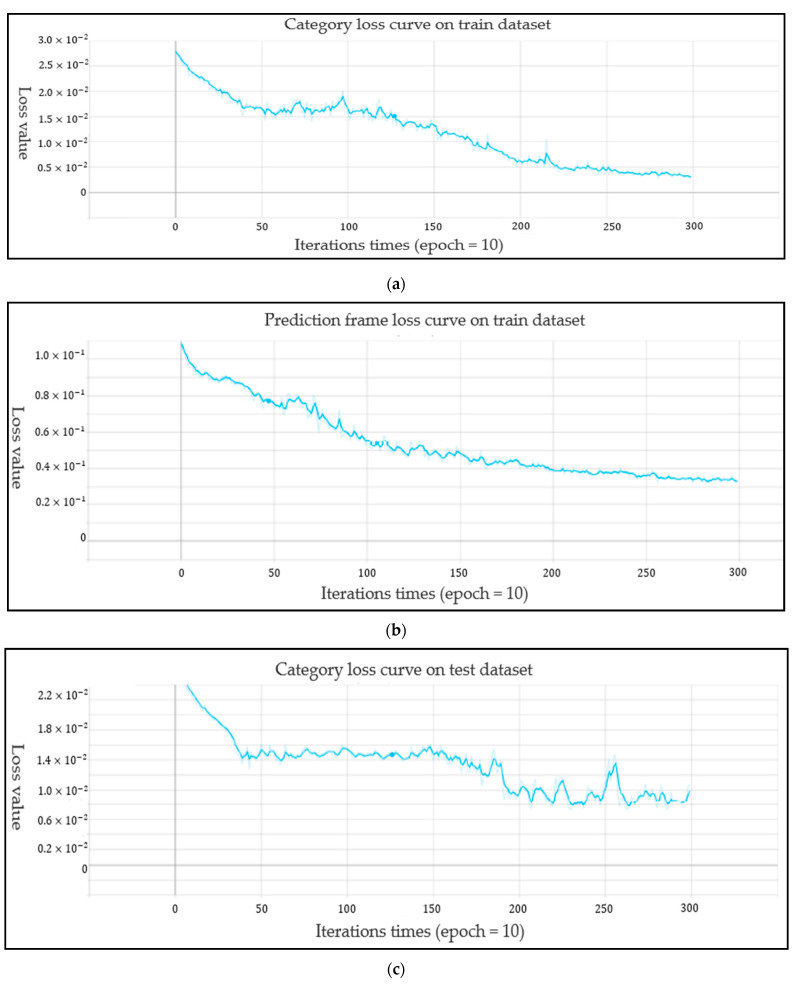

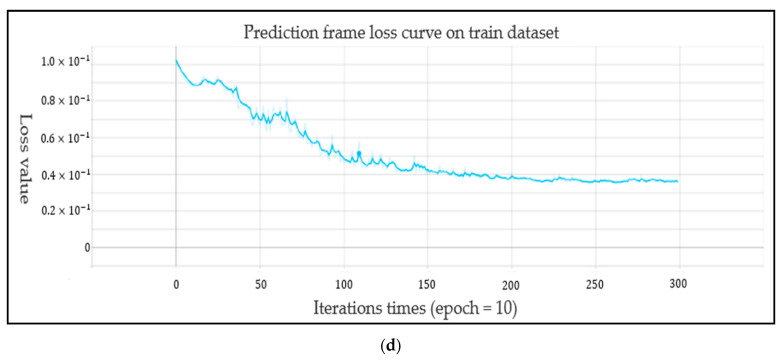
The loss curve of each data set of the Yolo-V4 model: (**a**) Motion category loss curve of train data; (**b**) Train set target frame loss curve; (**c**) Motion category loss curve of test data; (**d**) Test set target frame loss curve.

**Figure 12:**
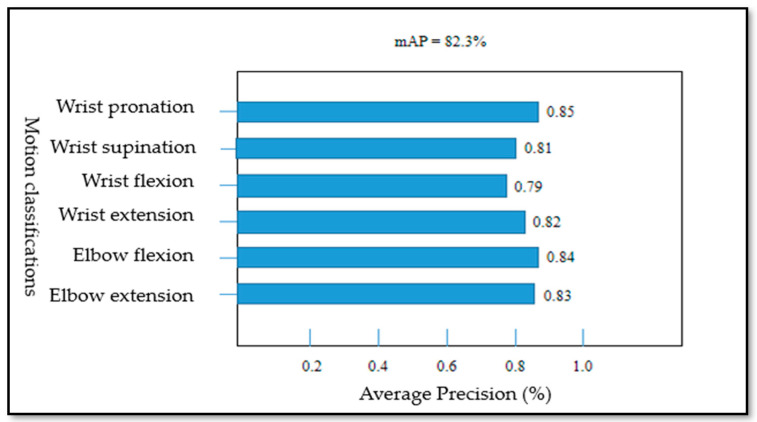
The mean Average Precision of each action.

For the performance of the Yolo-v4 network, the loss curves for each data set of the Yolo-V4 model are shown in Figure 11, Figure 11a shows the target frame loss curve of the training set, Figure 11b shows the category loss curve of the training set, Figure 11c shows the target frame loss curve of the test set, and Figure 11d shows the category loss curve of the test set. For the target detection algorithm, the loss curve cannot completely reflect the real situation of the model training process; however, the category loss can reflect the accuracy of classification. According to the loss curve, whether it is the regression loss of the target frame position or the action classification loss, the loss curve showed a downward trend and gradually tended to converge. Figure 12 shows the model mAP of six types of action recognition after 3000 iterations of training.

**Figure 13:**
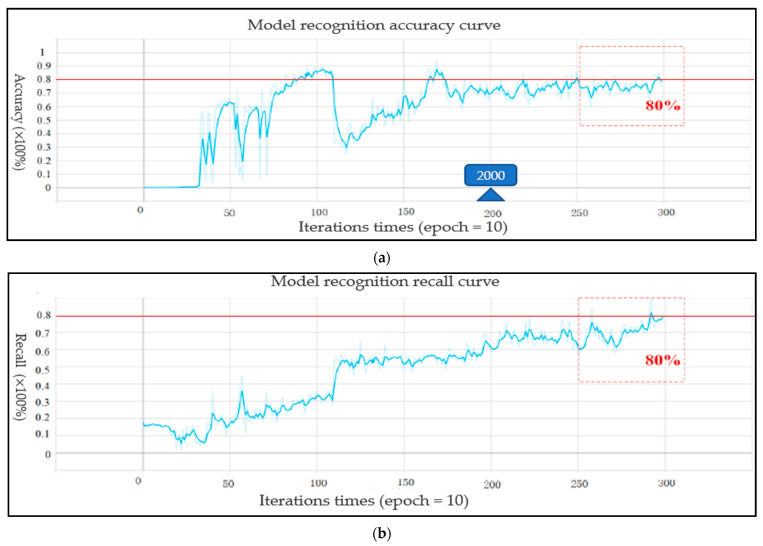
The precision and recall curves of the target detection network: (**a**) The precision curves; (**b**) The recall curves.

**Figure 14:**
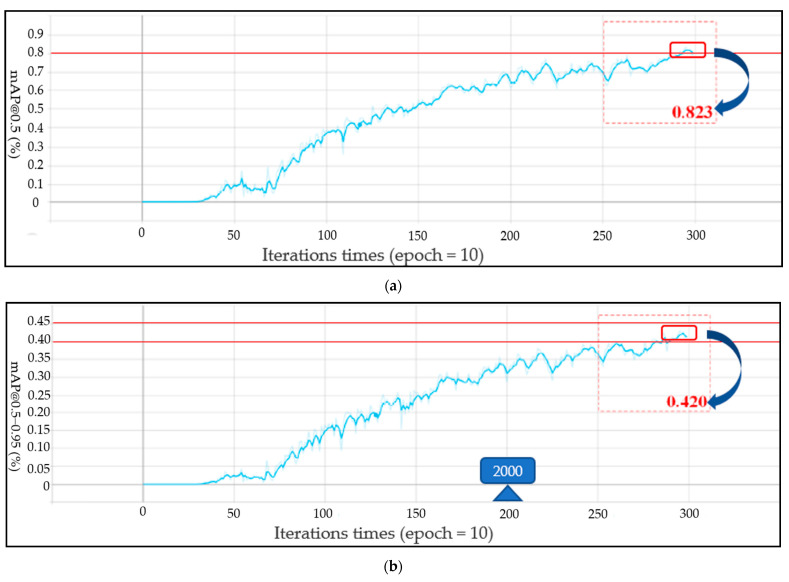
The mAP curve of target detection network performance: (**a**) The mAP@0.5 Curve; (**b**) The mAP@0.5–0.95 Curve curves.

**Figure 15:**
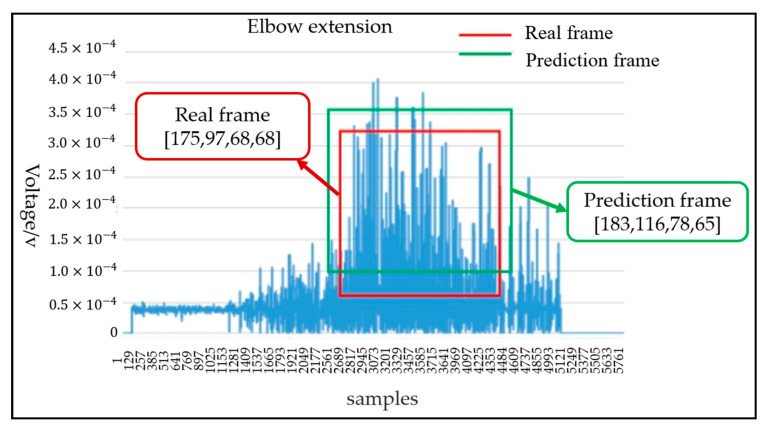
The proposed network prediction results of elbow extension on the validation data.

The mAP@0.5 of the proposed model can reach 0.823 and mAP@0.5–0.95 can reach 0.42. As shown in Figure 13a,b, the precision and recall curves of the target detection network are shown, respectively. When the training times of the model exceed 2000, the performance of the model exceeded 70%. With the increase of the training times, the accuracy gradually increased to 80%. The trend of the recall rate training curve also increased gradually with the increase of training times. When training 3000 times, the recall rate can reach more than 0.8. The Yolo model not only accurately identified the motion category, but also reduced the missed detection of the target. For the evaluation of target detectability, the recall rate and accuracy jointly determine the evaluation performance index map value. The mAP@0.5 and mAP@0.5–0.95 curves of two evaluation criteria are shown in Figure 14a,b, respectively. With the gradual increase of training times, when the training times approach 3000 epochs, mAP@0.5 will be above 0.82. After more than 2500 times of model training, mAP@0.5–0.95 gradually exceeded 0.4. Therefore, according to the mAP curve, the training model trained in this paper is available for the recognition of the upper limb joint based on sEMG signal images. The network model output of the Yolo-v4 algorithm included category prediction and maximum motion angle prediction. The performance of action category prediction was analyzed by mAP and the performance of maximum motion joint prediction was evaluated by Formula (4). The prediction result of motion category for one sEMG image from validation data was six (that is elbow extension) and the motion angle was calculated by the relevant target box and the real box. Figure 15 shows the prediction results of the proposed Yolo-V4 network are applied to the elbow extension movement on the verification data. The red box is the reference position (reference label array [175,97,68,68]) of the target in the picture. The blue box is the predicted position (prediction label array [183,116,78,65]) of the model in the picture. The ordinate value of the center point of the real frame was 97; the ordinate value of the center point of the prediction frame is 116; the maximum motion angle of the elbow was set to 135°. Since $h=97<h^{\prime }=116$, the predicted angle of the Yolo model for the extension motion of the elbow was 135°. Finally, the average recognition accuracy of the proposed Yolo-V4 model on the verification data can reach 80.7%. However, for the wrist joint, the model accuracy was reduced with 78 images that can be accurately identified per 100 images. The recognition accuracy of the elbow joint was 7–8% higher than that of the wrist joint, which can accurately identify 86 pictures per 100 pictures. The processing speed of the training model for the verification set was 17.97 MS for each picture, which could detect 56 pictures per second (that is, the recognition of 56 actions). The memory size occupied by the model does not exceed 39 MB. Table 7 shows the performance of the object detection Yolo algorithm on the verification set. Therefore, the target detection network proposed in this paper realized the action recognition strategy of upper limb rehabilitation training with fast detection speed and short response time.

## 4. Conclusions and Discussion

The purpose of this paper is to achieve a real-time target detection model for the upper limb motion classification and motion angle prediction with high accuracy and reliability.

For the upper limb exoskeleton rehabilitation, motion classification and motion angle prediction are two common research methods. Therefore, an improved upper limb intention prediction method was proposed in this paper, which can effectively achieve motion classification and maximum motion angle prediction simultaneously, especially for conventional low-density electrodes. Firstly, MGNet feature extraction network is trained based on multi-channel sEMG signal data, and efficient and accurate channels reduction and channel features fusion were realized through indicators such as accuracy and processing speed. Then, the fused sEMG signal image was input into the trunk feature extraction network MGNet, to be trained; its model weight was retained to obtain the feature extraction module in target detection. Finally, the Yolo-v4 target detection network was trained based on the fused sEMG data, and the upper limb rehabilitation motion recognition strategy was proposed. According to the experiment results, for the train data, the mAP@0.5 using the proposed model can reach 0.823, and mAP@0.5–0.95 can reach 0.42; For the verification data, the average recognition accuracy of the proposed Yolo-V4 model on the verification data can reach 80.7%. The processing speed of the training model for the verification set was 17.97 MS for each picture, which can detect 56 pictures per second (that is, the recognition of 56 actions). A support vector machine with kernel function “RBF”-based (SVM-RBF) sEMG signal classification technique was applied to compare the performance of the motion classification and joint angle prediction with the proposed method in validation dataset and train dataset. After the same sEMG signal preprocessing, time domain features, including integrated EMG (IEMG), mean absolute value (MAV), root mean square (RMS) were extracted from raw sEMG signals. As shown in Table 8, the comparison result of the classification performance and joint angle prediction are shown based on SVM and proposed method in validation dataset and train dataset. In terms of classification accuracy, SVM-based classification performance was better than that of the proposed method; however, there was no significant difference compared with the model recognition accuracy of this paper. The good feature extraction methods can improve the classification performance in practical application. However, it needs to be processed deeply to extract the most appropriate sEMG features and the huge amount of calculation. The balance between the estimation accuracy and real-time estimation speed should be considered. According to Table 7, the proposed is also meet the real-time requirements with high accuracy and reliability. However, the method proposed in this paper can also predict the angle of joint motion simultaneously. The proposed model in this paper will be applied to the low-density electrode exoskeleton rehabilitation robotic control. In the practical exoskeleton control, the angle signal can be mapped to the exoskeleton movement angle through corresponding control strategy. The angle signal can also be applied in dynamic model to calculate the joint torque of the exoskeleton. The prediction results in this paper can meet the requirements of these conventional applications. Meanwhile, the time delay caused by mechanical acquisition was reduced effectively based on the proposed model, thus realizing the real-time prediction of upper limb motion classifications. According to the results, the presented model can also be applied to motion recognition of the exoskeleton rehabilitation robotic. It is possible for the proposed method to predict the movement category and joint movement angle of upper limbs simultaneously with high accuracy and reliability in real-time. However, the actions in this paper are single action detection one by one and cannot be used in the detection and recognition of continuous action and composite motion, which is also the future research direction of this paper. For sEMG channel information fusion, this paper directly adopts the weighted linear summation method to ensure that the sum of channel weights is set as 1, which does not make analytical channel information fusion. In the future, the signal channel fusion method will be studied and analyzed to obtain better performance and the detection and recognition of continuous motion and compound motion will be studied.

## Figures and Tables

**Table 1:** Comparative experimental scheme and results of elbow motion channel reduction.

Experimental Scheme	Biceps Brachii	Triceps Brachii	Brachialis	Experimental Result	
				Accuracy (%)	Time Consuming (s)
Scheme 1	▲			61.9	103.29
Scheme 2	▲	▲		80.6	132.05
Scheme 3	▲	▲	▲	82.3	193.27

**Table 2:** Comparative experimental scheme and results of wrist pronation/external rotation channel reduction.

Experimental Scheme	Extensor Carpi Ulnaris	Flexor Carpi Radialis	Extensor Carpi Radialis	Flexor Carpi Ulnaris	Experimental Result	
					Accuracy (%)	Time Consuming (s)
Scheme 1	▲				69.7	97.93
Scheme 2	▲	▲			70.4	128.05
Scheme 3			▲		74.3	102.05
Scheme 4			▲	▲	75.1	130.57
Scheme 5	▲		▲		81.1	127.17
Scheme 6	▲	▲	▲	▲	81.9	193.27

**Table 3:** Comparative experimental scheme and results of wrist flexion/extension channel reduction.

Experimental Scheme	Extensor Carpi Ulnaris	Flexor Carpi Radialis	Extensor Carpi Radialis	Flexor Carpi Ulnaris	Experimental Result	
					Accuracy (%)	Time Consuming (s)
Scheme 1	▲				71.7	97.93
Scheme 2	▲	▲			72.5	128.05
Scheme 3			▲		64.9	101.93
Scheme 4			▲	▲	65.7	132.57
Scheme 5	▲		▲		80.3	131.53
Scheme 6	▲	▲	▲	▲	80.9	197.01

**Table 4:** The MGNet feature extraction network structure.

Input Size	Network Block	Expansion Factor (t)	Output Channels (c)	Number of Repetitions (n)	Step (s)
448 × 448 × 3	Conv2D	-	32	1	2
224 × 224 × 32	BottleNeck	1	16	1	1
224 × 224 × 16	BottleNeck	6	24	2	2
112 × 112 × 24	BottleNeck	6	32	3	2
56 × 56 × 32	Ghost	-	64	4	2
28 × 28 × 64	BottleNeck	6	96	3	2
14 × 14 × 96	BottleNeck	6	128	3	1
14 × 14 × 128	BottleNeck	6	160	3	2
7 × 7 × 160	BottleNeck	6	320	1	1
7 × 7 × 320	Conv2D 1 × 1	-	1280	1	1
7 × 7 × 1280	Avgpool 7 × 7	-	-	1	-
1 × 1 × 1280	Conv2D	-	K = 6	-	-

**Table 5:** The MGNet feature extraction network parameter.

Feature Extraction Layer	Input Size	Parameter Quantity	Model Size
3 × 3 × 3 × 32	448, 448, 3	864	-
dw 3 × 3 × 32	224, 224, 32	288	-
pw 1 × 1 × 32 × 16	224, 224, 32	512	-
pw 1 × 1 × 16 × (16 × 6)	224, 244, 16	1536	2
dw 3 × 3 × (16 × 6)	224, 224, (16 × 6)	864	2
pw 1 × 1 × (16 × 6) × 24	112, 112, (16 × 6)	2304	2
pw 1 × 1 × 24 × (24 × 6)	112, 112, 24	3456	3
dw 3 × 3 × (24 × 6)	56, 56, (24 × 6)	1296	3
pw 1 × 1 × (24 × 6) × 32	56, 56, (24 × 6)	4608	3
pc 1 × 1 × 32 × (64/2)	56, 56, 32	1024	4
co 3 × 3 × (64/2)	56, 56, 32	288	4
pw 1 × 1 × 64 × (64 × 6)	28, 28, 64	24,576	3
dw 3 × 3 × (64 × 6)	14, 14, (64 × 6)	3456	3
pw 1 × 1 × (64 × 6) × 96	14, 14, (64 × 6)	36,864	3
pw 1 × 1 × 96 × (96 × 6)	14, 14, 96	55,296	3
dw 3 × 3 × (96 × 6)	7, 7, (96 × 6)	5184	3
pw 1 × 1 × (96 × 6) × 160	7, 7, (96 × 6)	92,160	3
pw 1 × 1 × 160 × (160 × 6)	7, 7, 160	153,600	1
dw 3 × 3 × (160 × 6)	7, 7, (160 × 6)	8640	1
pw 1 × 1 × (160 × 6) × 320	7, 7, (160 × 6)	307,200	1
1 × 1 × 320 × 1280	1, 1, 320	409,600	-
1 × 1 × 1280 × 6	1, 1, 1280	7716	-
All	-	1,583,464	1.52 M-

**Table 6:** Performance comparison of different feature extraction networks.

Feature Extract Network Model	Accuracy(%)	Time Consuming (s)
MobileNet-V2	81.73	131.533
GhostNet	67.56	97.702
MGNet	81.91	109.392

**Table 7:** The performance of the training model on verification data.

	Processing Speed	Response Time	mAP
Verification data effect	1.7 s/900 images	17.97 ms for each image	80.7%

**Table 8:** The comparison of the classification performance and joint angle prediction based on SVM and proposed method in validation dataset and train dataset.

Methods	Best Accuracy		mAP		Angle Predict Error (°)	Predict
	Train	Validation	Train	Validation		
SVM-RBF	88.7%	85.8%	84.8%	83.6%	-	Only six motion classifications
Our methods	85.7%	82.4%	82.3%	80.7%	$0{}^{\circ}<\;|C_{pre\_angle}|<$ 10°	Six montion classifications and joint movement angle

## Data Availability

Not applicable.
